# Manifestations and implications of uncertainty for improving healthcare systems: an analysis of observational and interventional studies grounded in complexity science

**DOI:** 10.1186/s13012-014-0165-1

**Published:** 2014-11-19

**Authors:** Luci K Leykum, Holly J Lanham, Jacqueline A Pugh, Michael Parchman, Ruth A Anderson, Benjamin F Crabtree, Paul A Nutting, William L Miller, Kurt C Stange, Reuben R McDaniel

**Affiliations:** South Texas Veterans Health Care System, San Antonio, TX USA; University of Texas Health Science Center at San Antonio, San Antonio, TX USA; The University of Texas at Austin, Austin, TX USA; MacColl Center for Health Care Innovation, Group Health Research Institute, Seattle, WA USA; Duke University, Durham, NC USA; Department of Family Medicine, Robert Wood Johnson Medical School, University of Medicine and Dentistry of New Jersey, New Brunswick, NJ USA; Department of Family Medicine, University of Colorado School of Medicine, Aurora, CO USA; Lehigh Valley Health Network, Lehigh, PA USA; Case Western Reserve University, Cleveland, OH USA

**Keywords:** Complexity science, Relationships, Uncertainty, Healthcare systems, Implementation

## Abstract

**Background:**

The application of complexity science to understanding healthcare system improvement highlights the need to consider interdependencies within the system. One important aspect of the interdependencies in healthcare delivery systems is how individuals relate to each other. However, results from our observational and interventional studies focusing on relationships to understand and improve outcomes in a variety of healthcare settings have been inconsistent. We sought to better understand and explain these inconsistencies by analyzing our findings across studies and building new theory.

**Methods:**

We analyzed eight observational and interventional studies in which our author team was involved as the basis of our analysis, using a set theoretical qualitative comparative analytic approach. Over 16 investigative meetings spanning 11 months, we iteratively analyzed our studies, identifying patterns of characteristics that could explain our set of results.

Our initial focus on differences in setting did not explain our mixed results. We then turned to differences in patient care activities and tasks being studied and the attributes of the disease being treated. Finally, we examined the interdependence between task and disease.

**Results:**

We identified system-level uncertainty as a defining characteristic of complex systems through which we interpreted our results. We identified several characteristics of healthcare tasks and diseases that impact the ways uncertainty is manifest across diverse care delivery activities. These include disease-related uncertainty (pace of evolution of disease and patient control over outcomes) and task-related uncertainty (standardized versus customized, routine versus non-routine, and interdependencies required for task completion).

**Conclusions:**

Uncertainty is an important aspect of clinical systems that must be considered in designing approaches to improve healthcare system function. The uncertainty inherent in tasks and diseases, and how they come together in specific clinical settings, will influence the type of improvement strategies that are most likely to be successful. Process-based efforts appear best-suited for low-uncertainty contexts, while relationship-based approaches may be most effective for high-uncertainty situations.

**Electronic supplementary material:**

The online version of this article (doi:10.1186/s13012-014-0165-1) contains supplementary material, which is available to authorized users.

## Background

Results of efforts to improve healthcare systems remain inconsistent and disappointing. Despite significant investments to improve the areas of chronic disease management, quality improvement, and patient safety, consistent and sustained improvements in outcomes across settings have not been achieved [1]. In hospitals, ten years after the seminal IOM report on harm, data show little to no improvement in complications and adverse events [2],[3]. Data in outpatient settings have been mixed, with some major interventions showing relatively little impact on outcomes [4-6], others mixed [7], and some demonstrating system-wide improvement [8].

The research and clinical communities have begun applying different approaches to understanding healthcare systems with a goal of improving our ability to better understand and effectively change healthcare delivery. Examples include the application of manufacturing and engineering principles such as Six Sigma and Lean Management [9], as well as greater efforts to understand how local contexts influence outcomes of improvement initiatives [10]. In another approach, complexity science is being applied to healthcare settings [11-13]. Complexity science is a theoretical framework that provides the insight that systems are comprised of inter-related parts that interact in non-linear, potentially unpredictable ways [14],[15]. Individuals self-organize not necessarily according to hierarchy or organizational structure but based on how the work actually is accomplished [16-18]. System outcomes emerge from the interactions among elements of the system and from the local patterns of self-organization [19],[20]. These outcomes in turn create feedback loops that impact how the system evolves over time [16]. Self-organization, feedback loops, and the evolutionary nature of complex systems contribute to their unpredictability [20].

Our group has collaborated in a number of descriptive and interventional studies applying the complexity science framework to healthcare systems across a variety of settings. Our application of this framework focused on the interdependencies among individuals in healthcare delivery systems. [13],[14],[18],[19],[21],[22] Because of non-linearity and emergence inherent in complex systems, we wanted to look beyond reductionist or process-based approaches to understanding and improving healthcare systems and focus instead on how individuals relate, learn, make sense, and improvise as a way to understand and improve healthcare settings. The local self-organization inherent in complex systems also underscores the need to recognize and potentially leverage the relationships among individuals ‘on the ground’ [16],[17],[23]. We operationalized this focus on interdependencies in our studies in terms of understanding and influencing relationships among individuals. Thus, our studies have focused specifically on relational aspects of care delivery, such as the effects of relationships on learning and on leveraging relationships as a strategy to understand and improve processes of care and outcomes. Table 1 summarizes characteristics of complex system and how they have been applied in our work.

**Table 1:** **Characteristics of complex system and their application to our work**

Characteristic	Definition	Application
Individuals who learn	Individuals can process information and react to changes [23].	Focus on individuals in the system
Interconnections between agents	Individuals in the system are interconnected. Outcomes are the result of interactions across individuals rather than individual skill sets or behaviors [22].	Focus on how they relate, learn, and make sense
Self-organization	Order emerges from the interactions between individuals. These interactions cannot be completely understood or imposed from outside of the system [16].	Focus on patterns of relationships over time
Non-linearity and emergence	Complex behaviors emerge from simple rules. Inputs and outputs are not proportional or predictable [16]. System performance is not predictable over time [14],[15].	Focus on how individuals make sense of unexpected events and changes over time and learn from these experiences
Co-evolution and feedback loops	Individuals and microsystems are nested within other systems, which evolve and feed back over time [15],[21].	

Considered collectively, our studies produced inconsistent findings when we examined the associations between improving provider relationships, learning and a range of process and outcome measures in diverse settings, similar to the inconsistency in the improvement literature generally. In some of our studies, clear associations were found, but others showed mixed or no significant results. Additionally, even in studies with negative process or outcome results, clinic members reported improvements in their relationships or ability to learn, despite a lack of improvement in measured clinical outcomes. While it may not be a surprise that we observed unanticipated results while studying complex systems, these inconsistencies suggest that relational approaches to improvement may be more or less effective depending on the conditions or contexts, or for influencing certain outcomes.

In this analysis, we sought to better understand the conditions or contexts across which approaches of improving the relational aspects of care delivery as a strategy to improve processes and outcomes would be most effective. We believe this understanding is critical to our ability to improve healthcare system delivery, because interdependencies among individuals are a fundamental part of delivering care. We examined the results of eight studies in which we have been involved, systematically analyzing patterns of findings to provide new theoretical insights with regard to complexity and its manifestation in clinical settings. We then considered implications of our analysis for healthcare system intervention and improvement, exploring nuances of the roles of relationships, processes, and system resources on improving care. This analysis is not a comprehensive review of all studies seeking to improve healthcare outcomes by applying principles of complexity science. Instead, we use a set of studies in which we were involved and for which we have a rich understanding of the methods and findings. These insights allow us to build a theory [24] that will enable clinicians and researchers to more effectively intervene in healthcare systems to improve patient outcomes.

## Methods

We analyzed the group of studies in which any member of the research team participated as the potential set of studies to examine. Because of our many collaborations, at least two members of our group were involved in all chosen studies, and most typically involved three to five of the authors, giving us a robust shared knowledge of this body of work from which to start. Studies were purposefully selected to ensure a mix of study types (descriptive/observational and interventional), settings (primary care, inpatient care, skilled nursing care), and types of outcomes (process and health improvement) [4-6],[25-32]. Two studies were excluded from the sample we analyzed: one because it was an evaluation of the National Demonstration Project, an intervention to implement the patient-centered medical home model of care launched by the American Academy of Family Practice that was unrelated to our other work, and the other because it looked at relationships between and not only among practices and was duplicative with other studies in terms of setting and outcomes [33],[34]. This sampling approach afforded us a deep understanding of the research methods and findings, providing capacity to inform the development of new theory and recommendations for improvement efforts.

The observational and interventional studies and results from which we draw our observations are summarized in Table 2. Observational studies examined the association between relationships, learning, sensemaking, and improvising on patient outcomes. Interventional studies sought to change provider and staff relationships, learning, interdependencies, and interconnectedness as a strategy for improving patient outcomes. All studies are focused on healthcare providers, including physicians, nurses, clinic member staff, or ancillary services. Therefore, we use the term provider in a broad sense that would include all of these groups.

**Table 2:** **Studies examining the association between relationships and outcomes and their results**

***Observational studies***		
Study (PI)	Setting	Approach
Learning and Relationships in VA Primary Care Clinics (VA L&R, Pugh) [28]	19 Veteran Affairs primary care clinics in South Texas	N/A
Using Complexity Science to Understand Inpatient Microsystems (CS-IM, Leykum) [31],[32]	11 physician teams in two teaching hospitals in San Antonio	N/A
*Interventional studies*		
Study to Enhance Prevention by Understanding Practice (STEP-UP, Stange) [25-27]	Randomized trial 77 primary care practices in Ohio	Facilitation to enable process improvement to improved delivery of preventive services
Using Learning Teams for Reflective Adaptation study (ULTRA, Crabtree) [4]	25 primary care practices	Facilitated reflective adaptive process to improve team communication and adherence to clinical guidelines
Enhancing Practice, Improving Care (EPIC, Nutting and Crabtree) [6]	40 primary care practices	Three-arm study: Practice facilitation and CQI approaches to improve team relationships and diabetes care versus usual care group
ABC study (Parchman) [29]	40 primary care clinics in South Texas	One-year practice facilitation to improve relationships and diabetes outcomes
Supporting Colorectal Cancer Outcomes through Participatory Enhancements (SCOPE, Nutting and Crabtree) [5]	23 primary care practices in New Jersey	Six-month practice facilitation/learning collaboratives to improve screening rates
CONNECT (Anderson) [30]	Eight community and VA nursing homes (four interventions, four controls)	Improve information sharing across disciplines coupled with standard falls prevention intervention

### Analytic approach

Our analysis utilized the main effects papers from each of these studies. We used a set-theoretical approach in which we considered each study as a member of a number of sets with different attributes. More specifically, we used a qualitative comparative analytic (QCA) approach [35],[36] to analyze the findings from our studies as a collective, focusing specifically on uncovering patterns across studies regarding empirical linkage between relational aspects of care delivery and processes/outcomes. We held 16 multiple investigator discussions across an 11-month period, using a constant comparative approach [37] to iteratively define our subsets of studies, and reframe and refine our analyses. Our team has used this methodological approach in previous research [13],[38].

**Figure 1:**
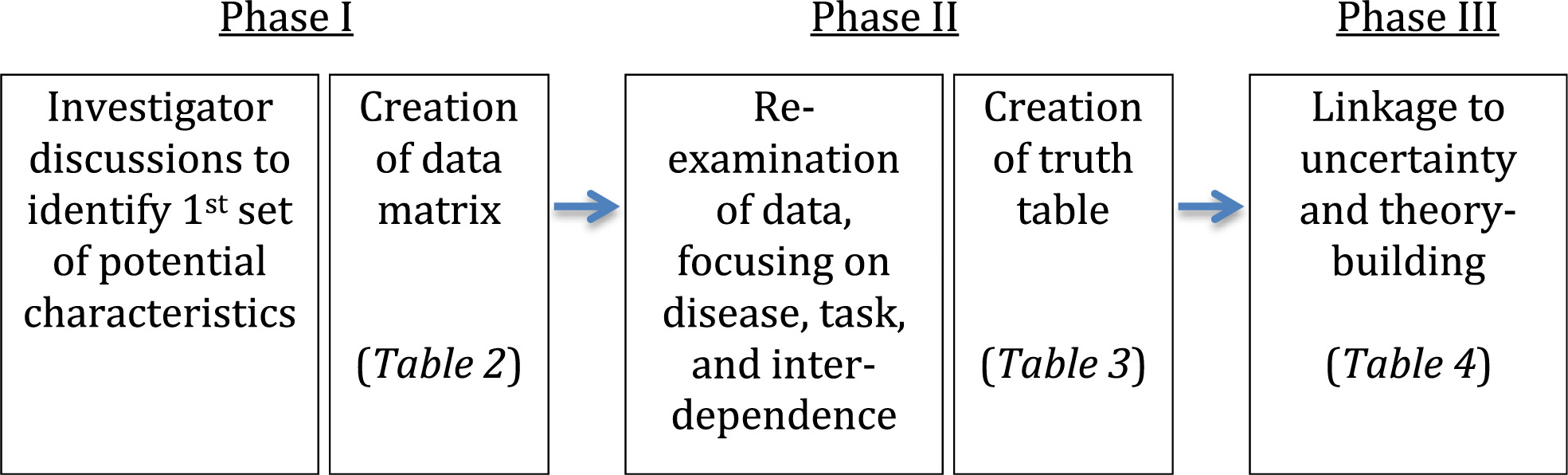
**Analytic approach.**

This iterative approach involved cycles of analysis, generating insights that systematically built on each other, as shown in Figure 1. Thus, results—or outcomes—from one set of discussions were often re-analyzed to yield a new set of updated insights. These cycles of systematic discussion, re-examination, and re-exploration involving the investigator team allowed us to continually re-interpret and refine our results. We built in strategies to guard against bias, such as having team members not involved in a particular study, and suggest contradictory interpretations of study results, continually seeking disconfirming evidence from the author team. We also included two researchers in our analysis meetings who were familiar with complexity science but were not involved in the primary studies, allowing us a further guard against bias.

Our small number of studies precluded an analysis of statistical significance. Instead, we used a QCA approach to guide our analysis of potential causality and construction of theoretical insights [36],[39]. We divided our studies into subsets based on their similarities, or sharing of specific attributes: for example, we grouped studies into sets based on setting or type of condition being studied. We then examined outcomes within these subsets, developing theoretical, causal explanations for similarities and differences based on attributes of the subsets. When outcomes within a subset of cases were similar, we considered the subset attributes to be potentially theoretically important or causal. When outcomes within a subset were different, we did not consider the subset attributes significant for the purposes of this analysis.

#### Analysis phase I: initial data matrix creation

Our initial attempts to classify our studies into sets centered on the characteristics of the studies, including setting and type of patient care activity. We included various settings because it initially appeared that studies in primary care settings were less likely to report improvements in process measures or outcomes. Our settings included primary care, inpatient medical care, and skilled nursing care. The types of patient care activities represented in our group of studies included preventive care, chronic disease management, and acute care. We considered patient care activities as potentially important because it appeared that relationships might have a stronger association with outcomes for certain types of care, such as acute care, rather than for other types of care such as prevention or chronic disease management. Because our studies had varied outcomes as a group, we classified the studies’ outcomes in terms of being relationship measures (this included relationships, learning, communication, and culture, and applied to interventional studies only), process measures, or clinical outcome measures.

The data matrix of this first classification is shown in Table 3. This table demonstrates that this classification did not consistently explain our pattern of positive or negative findings.

**Table 3:** **Initial data matrix considering setting and care delivery activities**

Study	Conditions		Results		
	Setting	Patient care activities	Relational	Process outcomes	Other outcomes
Learning and Relationships in VA Primary Care Clinics	Primary care	Preventive care (vaccination)	*Association* (patient experience)	No association (preventive care or chronic disease)	N/A
		Chronic disease management (hypertension (HTN), lipids, diabetes measures)			
Using Complexity Science to Understand Inpatient Microsystems	Inpatient	Acute medical care	N/A	N/A	*Improved* LOS, unnecessary LOS, complications
STEP-UP	Primary care	Preventive service delivery	N/A	*Improved*	N/A
ULTRA	Primary care	Team-wide communication	*Improved* (team communication)	No association	N/A
EPIC	Primary care	Diabetes, hypertension, lipid management	*Improved* (culture)	Improved in CQI group	No association (A1c, BP, lipids)
				No association in facilitation group	
ABC	Primary care	Reciprocal learning	*Improved* (learning, relational coordination)	N/A	*Improved* (ACIC, A1c)
SCOPE	Primary care	Colorectal cancer screening	N/A	No association	N/A
CONNECT	Nursing home	Safety culture	*Improved* (safety culture, communication)	No association	*Improved* fall rates

We next reconsidered the attributes of the clinical scenarios included in each of our studies. In this, we were influenced by our prior systematic reviews of organizational interventions to improve outcomes of patients with diabetes and congestive heart failure [13],[23]. In these analyses, we found that interventions that were more congruent with a complexity science approach were more likely to be effective, but the specific aspects of complexity science that were important in intervention success differed between diabetes and congestive heart failure. Thus, we looked at differences across diseases in our group of studies, identifying subsets based on types of diseases or illnesses. For example, we examined whether studies focused on rapidly progressing illnesses versus stable chronic diseases versus no illness at all. We also looked at the types of tasks, moving out of the general categories described above to consider task characteristics, such as whether the care being studied was broadly applicable or standardized across large groups or populations of patients or more customized to individuals. This distinction was grounded in part on the extent to which our studies in primary care implemented more standardized or customized interventions in terms of their application to patients.

Finally, we considered the interdependence, between setting, task, and disease (or lack of disease), or ways they might inter-relate. For example, while the setting alone might not be associated with strength of findings with regard to provider relationships, the setting might interact with task and disease in a way that the type of activity that is routine in the setting may be related to the importance of relationships as a strategy to improve care.

#### Analysis phase II: truth table creation

Thus, we identified disease, task, and their interdependence to be important theoretical variables for our analysis. In our discussions, we developed our understanding of these variables further, identifying pace of disease evolution and patient control over outcomes as key differences between the diseases included in our group of studies. With regard to clinical tasks, we identified the degree to which tasks were standardized versus customized to individual patients, the degree to which interdependencies among providers were required to accomplish the work, and the extent to which the task was routine or non-routine in a clinical setting. This latter consideration in particular spoke to the interdependence between disease, task, and setting.

Finally, we returned to uncertainty as our lens through which to explore differences in these attributes and their association with differences in study outcomes, using uncertainty as a key theoretical aspect of complex systems whose different manifestations might explain our findings. We hypothesized that tasks and diseases varied in the degree and type of uncertainty, and that this variation may also have influenced our range of findings. Interestingly, clinicians from each of the represented settings described that uncertainty was inherent in their work, and that there was a spectrum of highly uncertain and less uncertain work associated with each of the patient care activities. Thus, we sought a more nuanced way to understanding our pattern of findings. We considered the implications of these different manifestations of uncertainty in understanding and improving clinical systems, using this information as the basis for our analyses regarding the ways that variation in uncertainty is manifest. We developed a truth table (Table 4) to assess the applicability of the disease and task-related variables. In the truth table, we categorized each study where it best fits, though the categorizations may not be completely ‘clean.’ In our results, we develop these ideas further.

**Table 4:** **Truth table assessing the potential association between disease and task-related variables and whether provider relationships were associated with outcomes**

Disease-related		Task-related			Reported outcomes		Studies
Pace	Patient control	Standard/custom	Interdependency	Routine/non-routine	Process-outcomes	Other outcomes	
Slow	High	Standard	Low	Routine	No	Yes	L&R
Slow	High	Standard	Low	Mixed	Yes	-	STEP-UP, ABC
Slow	High	Standard	Low	Routine	No	-	EPIC, ULTRA, SCOPE
Slow	Low	Mixed	High	Mixed	-	Yes	CONNECT
Fast	Low	Customized	High	Non-routine	-	Yes	CS-IM

#### Analysis phase III: linkage to uncertainty

Because our studies were grounded in complexity science and we continue to apply this framework to understand healthcare systems, we turned to complexity science theory to interpret our pattern of results across studies. We came back to non-linearity and emergence as hallmark characteristics of complex systems. Emerging from these characteristics is the insight that system-level outcomes are often not predictable based on knowledge of systems’ components, and that knowledge and understandings of the structure and function of interdependencies in healthcare systems are required to improve outcomes. In complex systems, inputs and outputs are not proportional, and on a practical level, the relationship between inputs and outputs are not predictable. This lack of predictability, or uncertainty, is a defining characteristic of complex systems.

We considered degree of uncertainty to be a common theme across the disease and task-specific roles that could explain the variation we observed in our studies. We defined uncertainty as the unpredictability of what will happen. We operationalized uncertainty to refer to situations when the outcome cannot be controlled or predicted. In complex systems, not only might the specifics of the future be unknowable but also the future might differ greatly from what is expected. New outcomes or directions might emerge from the interaction of systems and system components. That said, by saying the future is not predictable, we do not imply that the future is random. On the contrary, patterns of trajectories in complex systems may be informative. For example, in clinical situations, we develop patient-specific scenarios using the patterns we see and update these scenarios depending on how the patterns unfold. Applying this to healthcare organizations, we are trying to identify the patterns in healthcare systems that enable us to understand when we need to pay attention to relationships, or relational aspects of care delivery.

## Results

Based on the results of our analysis of these eight studies, we identified characteristics of healthcare tasks and diseases that were associated with positive or negative outcomes in our studies of relational aspects of care delivery. Of particular importance was the characteristic of uncertainty in clinical situations. We explored the ways in which uncertainty must be considered in improvement efforts. In this process, we developed a new understanding of uncertainty in healthcare, summarized in Table 5 and detailed below. While our analysis demonstrated variability among tasks, diseases, and their interdependencies across clinical settings, we believe that patterns exist among this variability, and we can use these patterns to help recognize when it is particularly important to pay attention to how individuals in the system relate to each other.

**Table 5:** **Disease and task characteristics that influence uncertainty, and their manifestations in different clinical scenarios**

		Pace of disease evolution	Patient control over outcomes	Standardized versus customized	Routine versus non-routine	Work-sharing interdependency
*Clinical scenario*	Preventive care	Less rapid or not applicable, leading to less immediate uncertainty	May influence whether they access care	More standardized, less uncertain	More routine	Not reliant on other tasks, less uncertainty
	Chronic disease management	Typically less rapid. Exacerbations may develop in acute, atypical ways	Typically high, requiring patient adherence and engagement	Standardized delivery of recommended chronic care. Exacerbation care may have standardized and customized elements	More routine chronic care, exacerbation care may be routine and non-routine	High interdependence among specialties and settings
	Acute presentation of undiagnosed illness	Typically rapid	Lower	Workups may be mix of customized and standard, though some processes of care may be standard	Mixed	Multiple providers involved in care who are reliant on each other, many handoffs
	Sub-acute rehabilitation	Typically slow, with need for vigilance for clinical change	Varies	Routine daily care	Mixed	Multiple providers and handoffs, but fewer than inpatient settings

### Disease-related uncertainty

#### 1. Pace of evolution of the disease

The pace of evolution of care varies between outpatient, acute-care, and post-acute or nursing home settings. Primary care providers may follow an individual patient’s disease over years with occasional exacerbations or quick deteriorations that get transitioned to hospital settings or specialists’ offices. In the hospital, the pace of deterioration or recovery occurs much more quickly, and management of chronic diseases is often upended by the acute medical or surgical treatment. In nursing homes, both sub-acute and chronic care needs must be met. Prevention is important in all settings, though again the time frame over which preventive measures must occur varies.

Changes that develop quickly may be inherently more uncertain than those that develop slowly over time, in part because the shorter time course available to make decisions and assess impact. In this context, providers may be more reliant on relationships to successfully care for the patient. This may explain the significant impact of provider relationships on outcomes for hospitalized or nursing home patients versus chronic disease measures of care.

#### 2. Patient control over outcomes

The role of the patients and their degree of control over their own health outcomes vary tremendously between settings, illness, patients’ skills, patient support systems, and the interaction among these elements. In primary care settings, the amount of time the provider and the practice spends with patients is relatively small compared to the time patients spend managing their own diseases. In fact, one might argue that it is not even correct to use the word patient in this setting.

Greater patient control over outcomes may confer greater uncertainty from the perspective of the provider. Process measures of care for chronic disease are under the provider’s control to a much greater degree than chronic disease outcomes. In the former, the provider may only need to order tests or prescribe medications. Outcomes are dependent on the patient’s self-management and engagement in their disease process. Even though preventive care delivery may require action by the patient in terms of going for diagnostic testing, it is typically more of a one-time activity than a daily event embedded in their day-to-day lives.

This may explain the fact that we observe a lack of an association between relationships and process of care measures for chronic disease and preventive care, while we see positive results for an association between relationships and the patient experience of care and chronic disease outcomes. This may reflect the inherent greater uncertainty in the latter activities for which strengthening provider and patient relationships are important.

Differences in the degree of patient control over outcomes may also influence which relationships need attention. Relationships among providers may have less impact or may require different attributes in situations where the patient has a greater degree of control over outcomes. In these circumstances, provider-patient relationships may be more important. In the ABC study, we saw evidence that practice facilitation did lead some clinics to make changes in how they engaged and related to their patients, which may have led to positive findings. For example, a number of clinics started group patient visits, and one clinic started the ‘Under 7 club’ to engage their patients more fully in diabetes control.

In many situations in hospital and nursing home settings, patients are dependent and have less control over their management and outcomes, creating less uncertainty from the perspective of patient control over outcomes. In these cases, however, greater uncertainty from the perspectives of other aspects of care may exist. For this reason, provider relationships may be particularly important, and the impact of poor relationships among providers may be greater than when patients have greater control over outcomes. For example, effective care of a diabetes patient admitted to the hospital with diabetic ketoacidosis may be more contingent on provider relationships than more routine outpatient care of a diabetes patient.

### Task-related uncertainty

#### 1. Standardized versus customized activities

Uncertainty may also relate to the degree of standardization versus customization of care being delivered. Standardized care is relevant for populations of patients, which may make it inherently less uncertain in terms of its application to individuals. Customized care is focused on the individual for whom population guidelines do not exist or do not fit. It may involve working up problems that are not yet known or well understood or may involve tailoring current standards of care to individual patient needs. Customized care may be inherently less certain, as it is more tied to specific individual characteristics or manifestations that may be unknowable.

This distinction between standardized and customized care may explain some of the variance in our study results. We found a positive association between inpatient physician team relationships, unnecessary length of stay, and complications (CS-IM). These outcomes may require more customized care in terms of assessing individual post-acute care needs and preventing harm for patients most at-risk. Care in nursing homes may be quite individualized, leading to the positive associations we observed between relationships among caregivers and quality of care received by residents [39]. Conversely, many of our findings in primary care are related to prevention and did not demonstrate an association with relationships (Learning and Relationships in VA Primary Care Clinics (VA L&R) [28], Using Learning Teams for Reflective Adaptation (ULTRA) [4], Supporting Colorectal Cancer Outcomes Through Participatory Enhancements (SCOPE) [5], Enhancing Practice, Improving Care (EPIC) [6]), perhaps because these are more standardized aspects of care and less sensitive to relationship-based approaches. In the VA L&R study, relationships were significantly associated with the patient experience of care, perhaps a customized outcome.

#### 2. Work interdependencies required to deliver care

The degree to which care delivery falls within the realm of a single individual or service, or crosses individuals or services, also impacts uncertainty. Increasing the task interdependence in caring for a patient may also increase the uncertainty and need for relationship-centered approaches to improvement. For example, while there are evidence-based interventions that can be used to prevent falls, effective prevention still requires interaction and contribution from multiple nursing providers. In the CONNECT study to prevent falls in nursing homes, fall prevention was more successful in sites where the relationships were part of the intervention, with lower rates of falls over time.

Additionally, when care is dependent on multiple providers, the number and diversity of perspectives brought to the clinical situation may increase. To successfully bring these perspectives together into a shared approach, the relationship structure is critical. Finally, increased numbers of handoffs or transitions have been associated with adverse outcomes and are felt to be related to communication among providers. Handoffs may increase uncertainty and also appear to require a more robust relationship structure to manage effectively.

Several trends in healthcare are leading to increased emphasis and reliance on teams of providers delivering care, making this an important consideration in efforts to improve care delivery. One is the increasing numbers of care transitions and handoffs among providers in acute care settings, in part related to work-hour requirements for housestaff leading to less 24-hour consistent coverage. The advent of hospitalists and the unlikelihood of patients being followed by their primary care provider in the hospital is another trend that has increased work-sharing among individuals. The move towards interdisciplinary groups of professionals providing coordinated care in patient-centered medical homes may increase work-sharing among providers, particularly as more of the patient’s psychosocial needs are considered. Finally, changes in reimbursement such as bundled payments or shared savings require a different set of relationships among providers that promote coordination, communication, and shared understanding of management and treatment plans. These trends may lead to increases in work-sharing that increase uncertainty.

#### 3. Routine versus non-routine tasks

Caring for acutely ill patients with unusual illnesses or unusual manifestations of illness may confer a greater degree of uncertainty than managing more commonly seen disease situations. Again, this may lead to a greater reliance on relationships, in turn leading to a greater impact of the relationship infrastructure on patient outcomes. Completing more routine tasks may be less uncertain, and thus rely less on relationships. In primary care offices, delivery of preventive care or chronic disease care that is generally recommended may be inherently less uncertain than dealing with a new, undifferentiated complaint.

The degree of routine versus non-routine care, and its impact in terms of uncertainty, may also be context-dependent, and we must consider what is routine or not routine in specific clinical settings. A mismatch between the level of uncertainty inherent in the task and the types of tasks typically performed in the setting may lead to a greater degree of uncertainty. For example, providing initial care for a patient found to be in diabetic ketoacidosis in an outpatient setting may be more uncertain than providing that care in an emergency department, because the emergency department has routinized this type of care in a way that the typical primary care setting has not.

It may be possible to deliver care effectively across levels of task uncertainty within the same setting if an appropriate organizational structure is in place. For example, in many patient-centered medical home implementations, routine and preventive, low-complexity care is delivered by non-physician providers, while physicians focus on delivering care that is more highly uncertain. Thus, we must match the uncertainty of the work to be done with an organizational structure that can effectively navigate that level (or levels) of uncertainty.

## Discussion

Our analysis builds on the literature to date regarding uncertainty in healthcare or clinical situations. Uncertainty has been described in terms of illness or clinical progression, using terms such as ambiguity, inconsistency, vagueness, unpredictability, lack of information, and unfamiliarity [40]. A second way that uncertainty has been described is in terms of risk and risk assessment. In this approach, risk and uncertainty are often discussed as interchangeable, yet they differ in important ways [41]. A decision made under risk occurs when one can list all possible outcomes associated with a particular decision and assign a probability to each possible outcome. Managing risk is usually thought of as an information, or numeracy, activity where people have or can obtain the data required to support analyses for optimal decision-making. In contrast, uncertainty exists when one cannot list all possible outcomes or assign accurate probabilities to different outcomes. To manage risk, more information is generally effective, but the same is not true for managing uncertainty. When we discuss system uncertainty, we do not refer to situations where uncertainty exists solely because of lack of information. Instead, we refer to situations that are inherently unpredictable. Uncertainty can often be reduced with information, but it cannot be eliminated. Similarly, high-risk clinical situations in which outcomes may encompass life or death scenarios are not necessarily high uncertainty situations. For example, a critically ill patient may be high risk, but there may be relatively little clinical uncertainty.

More recently, Han et al. identified three ways that uncertainty is present in healthcare that expands the way we think about uncertainty: based not only on the unpredictable trajectory of patient illness, but also on the limits of scientific knowledge, and on system-level non-linearities [42]. While this taxonomy expands our conceptualizations of uncertainty in healthcare systems, it does not delve into the ways that these categories may vary in specific contexts. It also does not explicitly suggest strategies for navigating uncertainty or managing performance improvement in the face of these different types (sources) of uncertainty.

We defined uncertainty in terms of unpredictability. Our analysis suggests that uncertainty is an important aspect of clinical systems that must be considered in designing approaches to improve healthcare system function. The recognition of complexity in the delivery of healthcare provides the insight that improvement efforts must take uncertainty into account. Because uncertainty may vary depending on the disease or task and how they come together in specific settings, these interdependencies must be considered in intervention design. Understanding the patterns of task, disease, and the interdependencies among them in specific contexts that are associated with greater uncertainty will allow us to more effectively utilize relationally based approaches to improvement.

### Implications for interventions to improve healthcare delivery

To more effectively design interventions to improve patient outcomes, we propose approaching improvement in terms of impacting system interdependencies. These interdependencies include not only the processes of care and resources in the system, but also the relationship infrastructure among individuals in the system. The relative role of the uncertainty will vary as a function of the task, disease, the local context, and interdependence among them.

We propose considering improvement efforts in terms of changing the interdependencies in the system. These interdependencies include three elements: the resources available in the system; the processes utilized to accomplish work in the system; and the relational infrastructure among individuals in the system. The resources in the system will impact how the system functions and influence the approach taken to improvement efforts. For example, the physical layout of a clinic or inpatient unit will influence the communication patterns among providers [43]. The processes are the ways in which work is done in the system. These might include care pathways or protocols, or physical movement of individuals or materials throughout a system. Finally, the relationship infrastructure includes ways that providers relate to each other and to their patients. All of these aspects of a system influence each other. Resources will influence processes, processes influence ways that providers relate, and ways that people relate in turn influence processes and resource decisions. Finally, resources can be brought to bear to reinforce either processes or relationships in the system.

Implications of these differences in uncertainty for the role of process, resource, and relationship-based approaches for healthcare improvement are summarized in Table 6. We note that because any type of change leads to uncertainty, it may be helpful to consider the relational infrastructure and how individuals make sense and learn in any change effort, but targeting relationships as a key change intervention may not be necessary in low-uncertainty situations. Our focus in this work is not change efforts generally but rather on how varying manifestations of uncertainty in tasks, diseases, and settings being improved will influence the need for a focus on relationships in the intervention itself.

**Table 6:** **Implications of different levels of uncertainty for the role of process, relationships, and resources in improvement efforts**

Uncertainty	Process	Relationships	Resources
Low level	More likely to be effective. Consider quality/process improvement approaches that are generally applicable.	Less likely to be more effective than process-based interventions. Consider only as additive/enhancing for process-based interventions.	Consider in terms of supporting processes, e.g., deploying system-wide pathways or standardized protocols through an electronic health record.
High level	Less likely to be effective, or sufficient to enable necessary change.	More likely to be required for successful change. Consider approaches such as huddles, facilitation, or collaboratives.	Consider in terms of need for human or other resources required to enable sensemaking, e.g., care coordinators integrated with other providers for high-utilizer patients.

#### Low levels of uncertainty

Delivery of preventive care, recommended chronic disease management, and guideline-concordant population-based care seem to be relatively standardized, routine, low-uncertainty activities. Process-based interventions may be most useful in these circumstances in which the target of improvement is one that is applicable to almost all patients. For example, in primary care settings, urine screening for microalbuminuria in diabetic patients is recommended for all diabetic patients and may be well-suited to process-based interventions such as clinical reminders, automated order sets, or clinical protocols. Resource allocation in these contexts may focus on infrastructure that improves access to care, or implementation of technologies to improve guideline-concordant care. The delivery of preventive and chronic disease care in the VA illustrates the effectiveness of process-based interventions on routine care delivery. The VA has made considerable investment in clinical reminders and other processes that put delivery of preventive and chronic disease care at the forefront of the primary care delivery system. Our own results in VA primary care show that markers of preventive and chronic disease care were high and not associated with between-clinic differences in provider and staff relationships. Similarly, the SCOPE trial, changes in clinic members’ learning did not lead to differences in screening rates.

Low uncertainty situations may have high clinical risk. For example, trauma patients are high-risk for mortality, but well-established protocols that are generally applicable guide initial assessment and care. In these types of low uncertainty but high-risk situations, processes of care that ensure that all patients receive recommended care are critical.

#### High levels of uncertainty

Paying attention to provider relationships may be more important in settings where there is a higher level of uncertainty, making improvement through the application of processes or resources alone less effective. These circumstances include those where there is a greater need to share work and where clinical issues are non-routine, customized, and quickly evolving. In nursing homes, a requirement that staff continuously respond to individual needs of very diverse residents may lead to situations with high levels of uncertainty. In hospital settings, treatment of the patient often occurs without stopping. This leads to a focus on assessments, handoffs, and transitions that involve many providers across and within specialties. In these contexts, the ways that the providers relate and make sense are critical to good outcomes, in turn requiring a relationship infrastructure that enables effective communication. This need to distribute care across providers may not occur to the same degree in primary care settings, particularly in the delivery of routine or preventative services.

Alternatively, managing the workup or treatment of a patient among multiple providers in the primary care setting, or to and from another setting to primary care, may be quite complex. The greater the requirement for coordination or care sharing among providers, the more impact the provider relationships will have on patient outcomes. In these circumstances, resources might be better deployed to improve the ways that providers relate to each other and make sense of non-routine issues. For example, the introduction of navigators or care coordinators might be an effective approach to supporting the ways that providers relate with each other and their patients by opening a new channel of communication that can support patients’ and providers’ ability to make sense and learn.

High uncertainty situations require an adaptive approach. Adaptive problems require more emphasis on relationships, as well as how providers make sense of what is happening [21],[44], improvise [45], and learn [46-48]. Improving care for chronically ill patients who interact frequently with the healthcare system for acute care services exemplifies the ways in which uncertainty might influence the types of approaches that are most likely to be effective. Some aspects of care, such as periodically recommended reassessment of ejection fraction, nutritional counseling, or weight monitoring, are more standardized and routine in outpatient than in acute settings. Efforts to improve those more routine aspects of care are well-suited to process-based approaches such as reminders or decision support, or resource-based approaches of adding nutritional education resources or home monitoring support. However, patients have a high degree of control over outcomes, and improving the relationships between the patient, family, and providers are likely to be important for optimal self-management. Acute exacerbations can be unpredictable, and thus their workup will likely require some customization and coordination among providers that is dependent on provider relationships and sensemaking. Finally, once admitted, patients with heart failure are at high risk for readmission, and successful transitions to home are likely to require some customization of discharge plans for individual patients. In this example, uncertainty manifests differently in different aspects of heart failure care delivery, and improving outcomes for heart failure patients requires attention to the interdependencies that are required for improvement.

### Limitations

While we had a diverse sample of studies conducted across a number of healthcare settings, our studies were predominantly conducted in primary care settings, and not all settings were represented. However, our approach provided us with rich data that informed our analysis that could not be obtained from a less in-depth approach. Our primary care studies also did not examine issues of multi-morbidity or care transitions between settings. Our framework for considering uncertainty based on the interplay between disease, task, and setting would apply to those issues.

Additionally, relationships have multiple aspects and characteristics. These various characteristics may also have different degrees of importance in improvement interventions based on the task, disease, or setting. We do not explore those potential nuances, but they are important areas of further development.

## Conclusions

Recognizing healthcare systems as complex systems highlights the uncertainty and unpredictability inherent in healthcare delivery. It also highlights the patterns of uncertainty that exist. Uncertainty has been described in terms of system non-linearities, limits of scientific knowledge, and unpredictable trajectories of disease. This paper adds to the literature on uncertainty in healthcare systems by developing an empirically grounded approach to understanding how patterns of uncertainty might vary depending on the task being done, disease being treated, or setting in which care is delivered, leading to low or high uncertainty situations. Pace of evolution of disease and degree of patient control over outcomes may be ways to consider the unpredictable trajectory of disease and the limits of scientific knowledge. While all diseases have some level of unpredictability in their trajectories, customized and non-routine care may have the greatest. Task-related uncertainties may be examples of the types of uncertainty inherent in the system.

Our analyses have implications for efforts to improve healthcare system performance and patient outcomes. Understanding differences in the ways that uncertainty is manifest in different clinical scenarios will lead to an improved understanding of the types of improvement efforts that will be most likely to be effective. Differences in uncertainty levels based on task, disease, setting, and their interdependence should be considered when selecting improvement strategies in healthcare. Recognizing patterns of how task, disease, and their interdependence come together from the perspective of uncertainty will afford a greater ability to understand local patterns of self-organization and recognize when paying attention to the relational aspects of care delivery will be critical for intervention success [11]. In these cases, fostering sensemaking, learning, and improvising could be important strategies for improvement. For example, understanding the degree to which a change impacts routine versus non-routine care or requires the sharing of work across multiple providers may be helpful in deciding what approaches are most likely to be effective. Trying to improve non-routine care with only process-based interventions may not be as successful as an intervention based on reshaping the relationships among providers. Being more deliberate about these interdependencies and their role will lead to improved interventions, particularly in the context of reimbursement and policy changes that promote effective coordination and communication among providers. Better matching of improvement strategies to the nature of the system improvement will increase the likelihood of success.

## Authors’ original submitted files for images

Below are the links to the authors’ original submitted files for images. Authors’ original file for figure 1 Authors’ original file for figure 2
