# Supplementary figures and images for: Manifestations and implications of uncertainty for improving healthcare systems: an analysis of observational and interventional studies grounded in complexity science
Source: Implement Sci. 2014 Nov 19;9:165. doi: 10.1186/s13012-014-0165-1 (PMC4239371; doi:10.1186/s13012-014-0165-1)

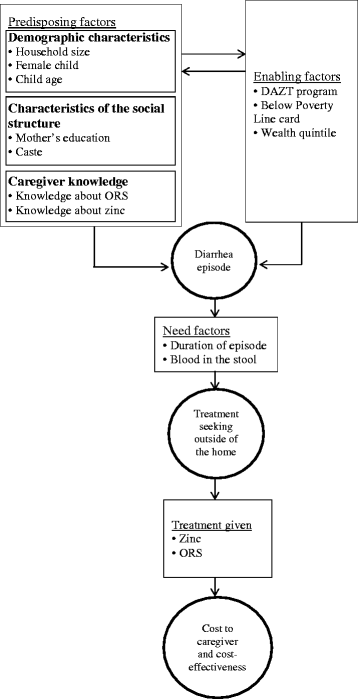

Supplement: Supplementary file 1 — Authors’ original file for figure 1 [file 13012_2014_165_MOESM1_ESM.gif]
