# Supplementary material for: Manifestations and implications of uncertainty for improving healthcare systems: an analysis of observational and interventional studies grounded in complexity science
Source: Implement Sci. 2014 Nov 19;9:165. doi: 10.1186/s13012-014-0165-1 (PMC4239371; doi:10.1186/s13012-014-0165-1)
Supplement: Supplementary file 2 — Authors’ original file for figure 2 [file 13012_2014_165_MOESM2_ESM.docx]

**Figure 2: Uncertainty, improvement strategies, and patient outcomes**

Setting characteristics

Sensemaking, improvising, learning

Patient

outcomes

Improvement

strategy

Uncertainty

level

Task

characteristics

Disease

characteristics
